# Supplementary material for: Predictive Potential of Cmax Bioequivalence in Pilot Bioavailability/Bioequivalence Studies, through the Alternative ƒ2 Similarity Factor Method
Source: Pharmaceutics. 2023 Oct 20;15(10):2498. doi: 10.3390/pharmaceutics15102498 (PMC10610255; doi:10.3390/pharmaceutics15102498)
Supplement: Supplementary file 1 [file pharmaceutics-15-02498-s001.zip › pharmaceutics-2614531-supplementary.pdf]

Predictive Potential of Cmax Bioequivalence in Pilot Bioavailability/Bioequivalence Studies, Through the Alternative f2 Similarity Factor

Sara Carolina Henriques, Paulo Paixão, Luís Almeida, Nuno Elvas Silva

Supplementary Material
Descriptive Statistics of Simulated Pharmacokinetic Data

Contents

S.1. Simulations Where Test Product Presents a Lower Bioavailability than the Reference Product 3

S.1.1. Simulated Volume of Distribution . . . 3

S.1.2. Simulated Plasma Concentrations . . . 7

S.1.3. Simulated Non-Compartmental Analysis Parameters . . . 11

S.1.3.1. Maximum Observed Concentration . . . 11

S.1.3.2. Time of Maximum Observed Concentration . . . 14

S.1.3.3. Area Under the Concentration-Time Curve . . . 16

S.2. Simulations Where Test Product Presents a Higher Bioavailability than the Reference Product 19

S.2.1. Simulated Volume of Distribution . . . 19

S.2.2. Simulated Plasma Concentrations . . . 23

S.2.3. Simulated Non-Compartmental Analysis Parameters . . . 27

S.2.3.1. Maximum Observed Concentration . . . 27

S.2.3.2. Time of Maximum Observed Concentration . . . 30

S.2.3.3. Area Under the Concentration-Time Curve . . . 32

S.1. Simulations Where Test Product Presents a Lower Bioavailability than the Reference Product

S.1.1. Simulated Volume of Distribution

Table S1: Summary Statistics of Simulated Volume of Distribution, for Simulations where Test Product Presents a Lower Bioavailability than the Reference Product

	True Bioequivalent		True Bioinequivalent	
	Reference	Test	Reference	Test
30% IIV & 0% IOV	58.79 (30.7%) [58.72–58.87]	58.79 (30.7%) [58.72–58.87]	58.64 (30.5%) [58.57–58.72]	58.64 (30.5%) [58.57–58.72]
30% IIV & 10% IOV	58.95 (32.2%) [58.87–59.03]	58.95 (32.2%) [58.87–59.03]	58.93 (32.4%) [58.85–59.01]	58.93 (32.4%) [58.85–59.01]
30% IIV & 20% IOV	58.67 (37.1%) [58.58–58.76]	58.65 (37.2%) [58.56–58.74]	58.63 (37.6%) [58.53–58.72]	58.64 (37.6%) [58.55–58.74]
30% IIV & 30% IOV	58.71 (44.4%) [58.6–58.82]	58.76 (44.3%) [58.66–58.87]	58.85 (44.1%) [58.74–58.95]	58.88 (44%) [58.77–58.98]
30% IIV & 40% IOV	58.83 (53.4%) [58.71–58.96]	58.81 (53.2%) [58.68–58.93]	58.86 (53.1%) [58.73–58.98]	58.77 (52.9%) [58.65–58.9]
30% IIV & 50% IOV	58.93 (63.1%) [58.78–59.07]	59.11 (63.4%) [58.96–59.26]	58.77 (63.5%) [58.63–58.92]	58.73 (63.5%) [58.59–58.88]
30% IIV & 60% IOV	58.2 (75.3%) [58.04–58.37]	58.5 (75.8%) [58.34–58.67]	58.67 (75%) [58.5–58.84]	58.77 (74.8%) [58.6–58.93]

Values for Volume of Distribution (V) are in liters (L), and are presented as geometric mean (G_{mean}), with geometric coefficient of variation (GCV%) within parenthesis, and 95% confidence interval (CI) of the G_{mean} within square brackets.

$N = 210000$ for each group.

IIV – Inter-individual variability; IOV – Inter-occasion variability.

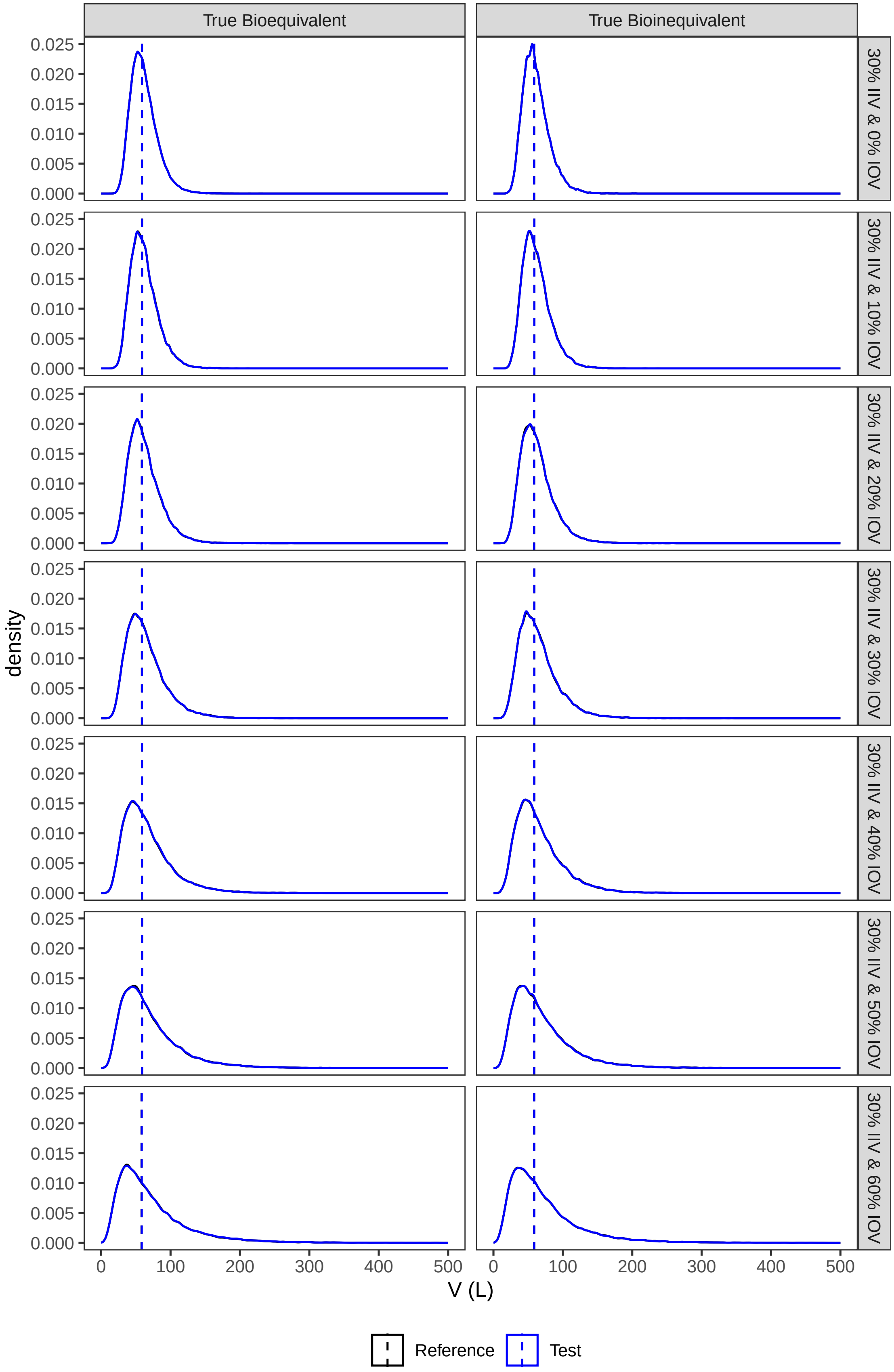

Figure S1: Distribution of Simulated Volume of Distribution, for Simulations where Test Product Presents a Lower Bioavailability than the Reference Product

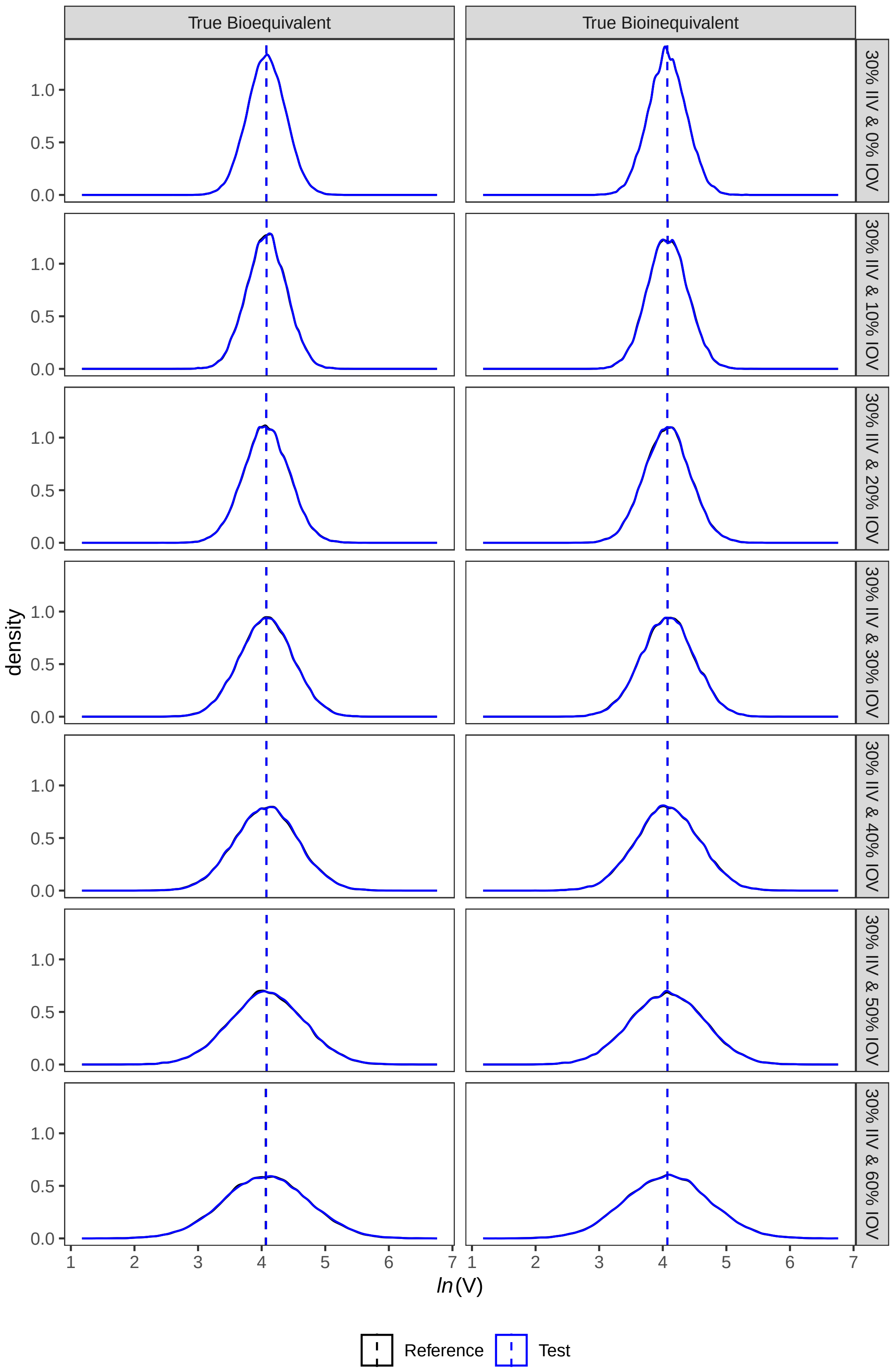

Figure S2: Distribution of *Ln*-Transformed Simulated Volume of Distribution, for Simulations where Test Product Presents a Lower Bioavailability than the Reference Product

S.1.2. Simulated Plasma Concentrations

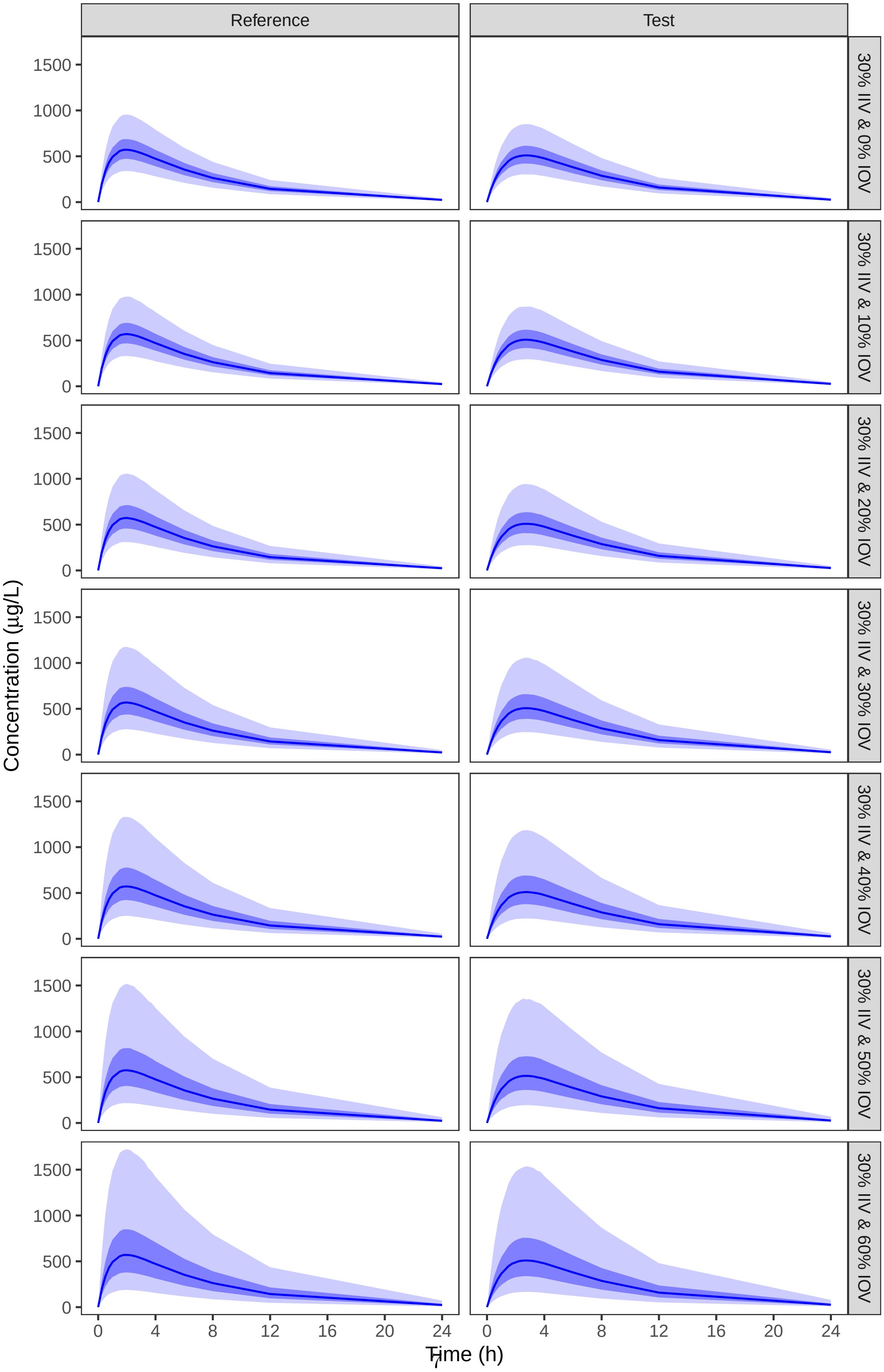

Figure S3: 90% Confidence Interval of Simulated Concentration-Time Data, for True Bioequivalent Simula-tions where Test Product Presents a Lower Bioavailability than the Reference Product – Linear Scale

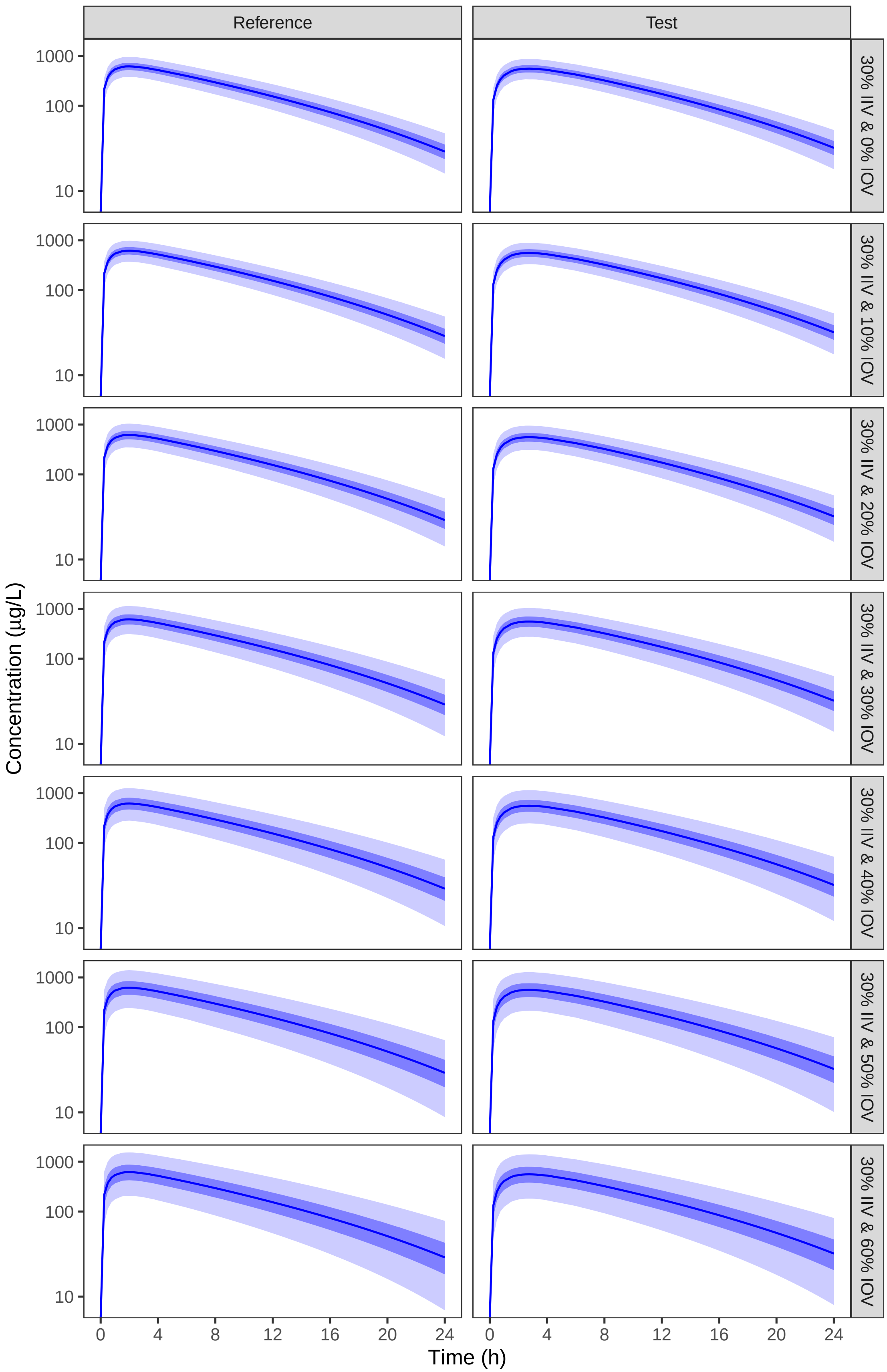

Figure S4: 90% Confidence Interval of Simulated Concentration-Time Data, for True Bioequivalent Simula-tions where Test Product Presents a Lower Bioavailability than the Reference Product – Semi-Logarithmic Scale

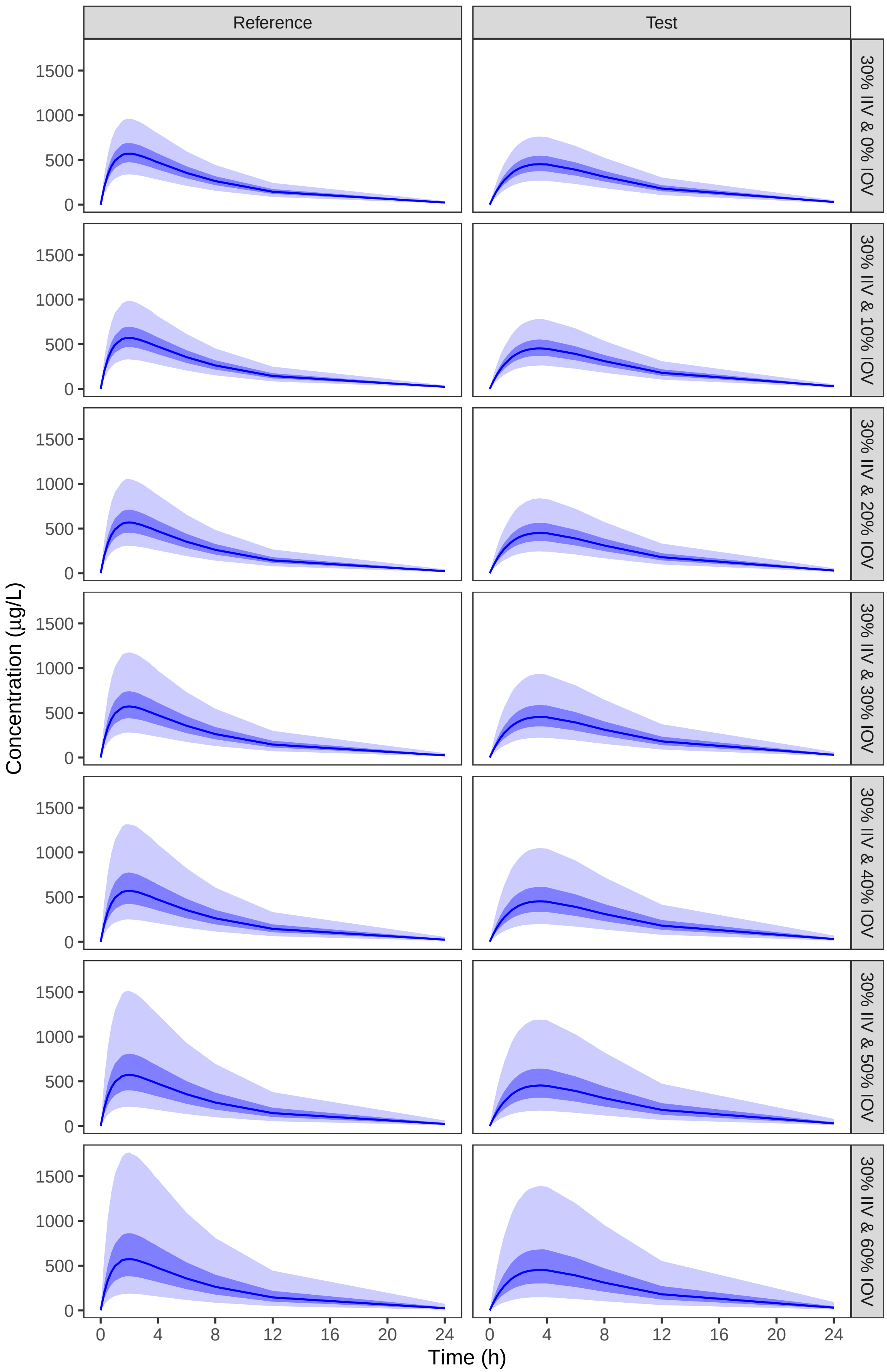

Figure S5: 90% Confidence Interval of Simulated Concentration-Time Data, for True Bioinequivalent Simulations where Test Product Presents a Lower Bioavailability than the Reference Product – Linear Scale

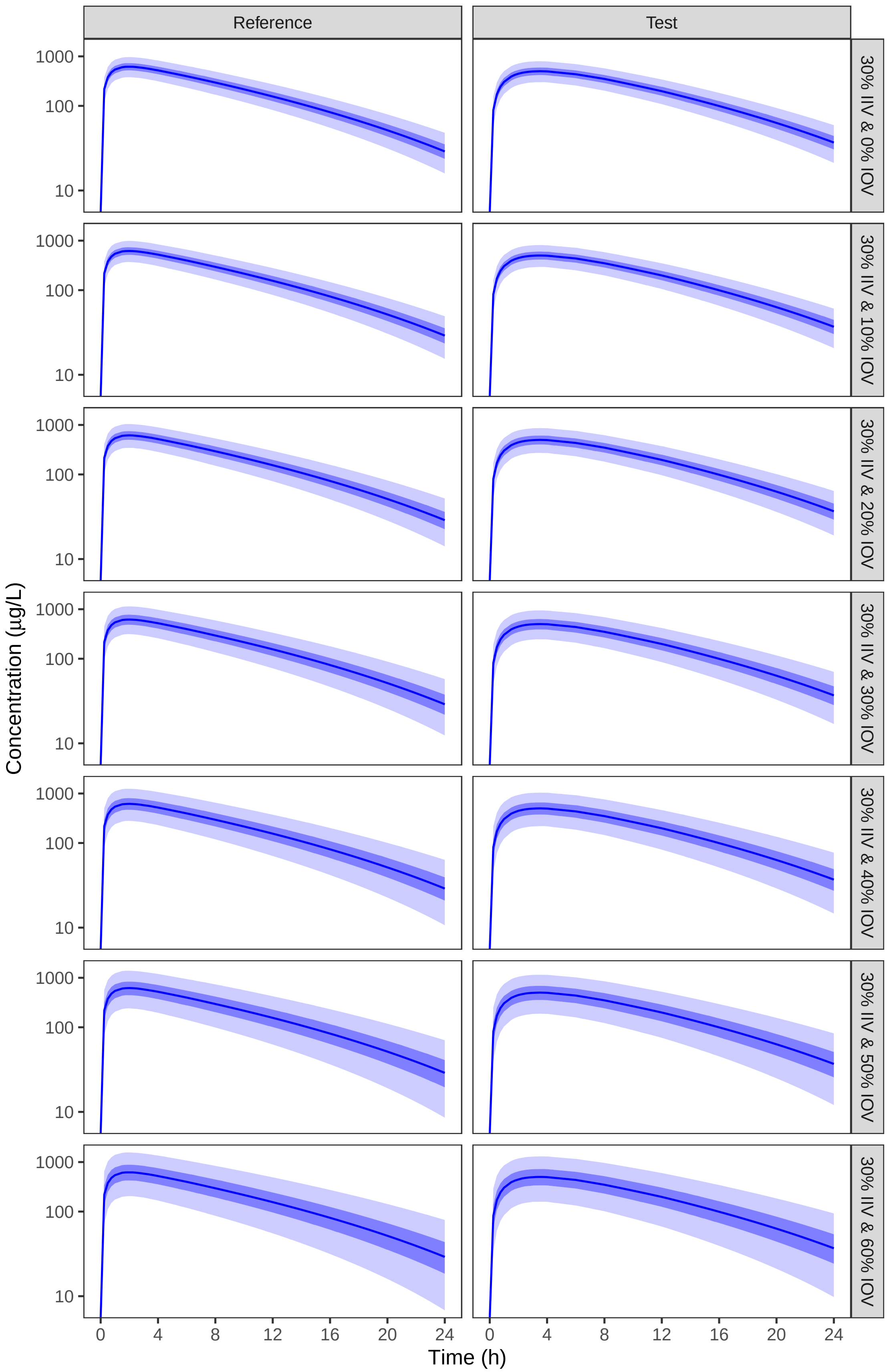

Figure S6: 90% Confidence Interval of Simulated Concentration-Time Data, for True Bioinequivalent Simula-tions where Test Product Presents a Lower Bioavailability than the Reference Product – Semi-Logarithmic Scale

S.1.3. Simulated Non-Compartmental Analysis Parameters

S.1.3.1. Maximum Observed Concentration

Table S2: Summary Statistics of Simulated Cmax, for Simulations where Test Product Presents a Lower Bioavailability than the Reference Product

	True Bioequivalent		True Bioinequivalent	
	Reference	Test	Reference	Test
30% IIV & 0% IOV	642.57 (31.4%) [641.73–643.41]	580.24 (31.3%) [579.48–581]	643.6 (31.1%) [642.76–644.44]	514.58 (31.2%) [513.91–515.25]
30% IIV & 10% IOV	640.65 (32.9%) [639.78–641.53]	578.51 (32.9%) [577.72–579.3]	640.92 (33%) [640.03–641.8]	512.06 (33%) [511.36–512.77]
30% IIV & 20% IOV	643.95 (37.7%) [642.94–644.95]	581.93 (37.7%) [581.03–582.84]	643.91 (38.2%) [642.9–644.93]	514.8 (38.1%) [513.99–515.61]
30% IIV & 30% IOV	643.26 (44.8%) [642.08–644.43]	580.42 (44.9%) [579.36–581.48]	642 (44.6%) [640.83–643.17]	512.7 (44.5%) [511.77–513.64]
30% IIV & 40% IOV	642.05 (53.8%) [640.67–643.44]	579.77 (53.6%) [578.53–581.02]	641.45 (53.6%) [640.07–642.83]	513.71 (53.5%) [512.6–514.81]
30% IIV & 50% IOV	640.86 (63.5%) [639.27–642.46]	576.84 (63.7%) [575.4–578.28]	642.08 (64%) [640.48–643.69]	513.91 (63.9%) [512.63–515.2]
30% IIV & 60% IOV	648.75 (75.8%) [646.88–650.62]	582.9 (76.1%) [581.22–584.59]	643.82 (75.5%) [641.98–645.67]	514.29 (75.2%) [512.82–515.76]

Values for Maximum Observed Concentration (C_{max}) are in μg/L, and are presented as G_{mean}, with GCV% within parenthesis, and 95% CI of the G_{mean} within square brackets.

$N = 210000$ for each group.

IIV – Inter-individual variability; IOV – Inter-occasion variability.

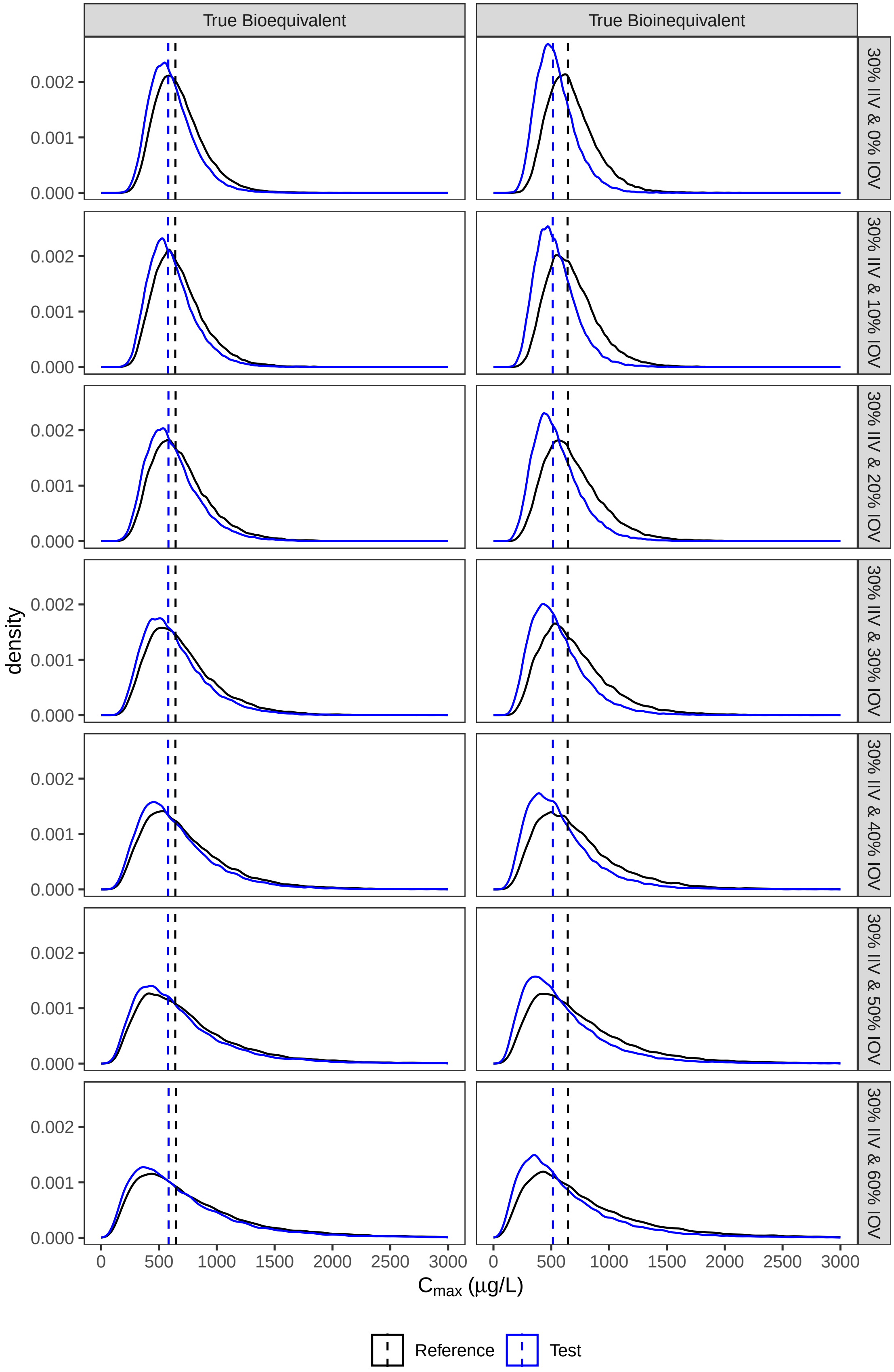

Figure S7: Distribution of Observed Cmax, for Simulations where Test Product Presents a Lower Bioavail-ability than the Reference Product

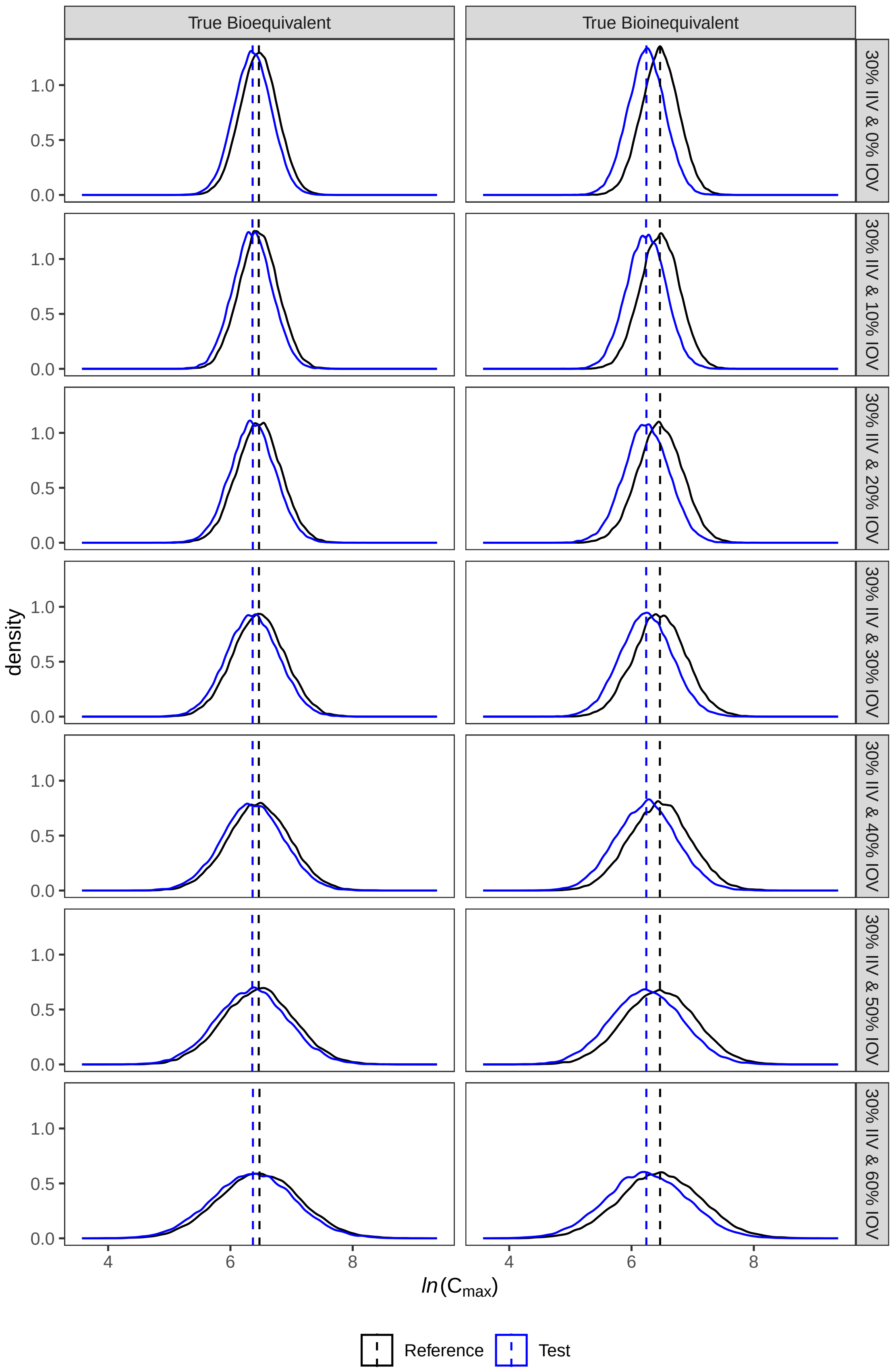

Figure S8: Distribution of *Ln*-Transformed Observed Cmax, for Simulations where Test Product Presents a Lower Bioavailability than the Reference Product

S.1.3.2. Time of Maximum Observed Concentration

Table S3: Summary Statistics of Simulated Cmax, for Simulations where Test Product Presents a Lower Bioavailability than the Reference Product

	True Bioequivalent		True Bioinequivalent	
	Reference	Test	Reference	Test
30% IIV & 0% IOV	2.25 (0.75–4)	2.75 (1–6)	2.25 (0.75–4)	3.25 (1.5–6)
30% IIV & 10% IOV	2.25 (0.75–4)	2.75 (1.5–6)	2.25 (0.75–4)	3.25 (1.5–8)
30% IIV & 20% IOV	2.25 (0.75–4)	2.75 (1–6)	2.25 (0.75–4)	3.25 (1.5–8)
30% IIV & 30% IOV	2.25 (0.75–4)	2.75 (1–6)	2.25 (0.75–4)	3.25 (1.5–6)
30% IIV & 40% IOV	2.25 (0.75–4)	2.75 (1–6)	2.25 (0.75–4)	3.25 (1.5–6)
30% IIV & 50% IOV	2.25 (0.75–4)	2.75 (1–6)	2.25 (0.75–4)	3.25 (1.5–8)
30% IIV & 60% IOV	2.25 (0.75–4)	2.75 (1.5–6)	2.25 (0.75–4)	3.25 (1.5–8)

Values for Time of Maximum Observed Concentration (t_{max}) are in hours (h), and are presented as median with range within parenthesis.

$N = 210000$ for each group.

IIV – Inter-individual variability; IOV – Inter-occasion variability.

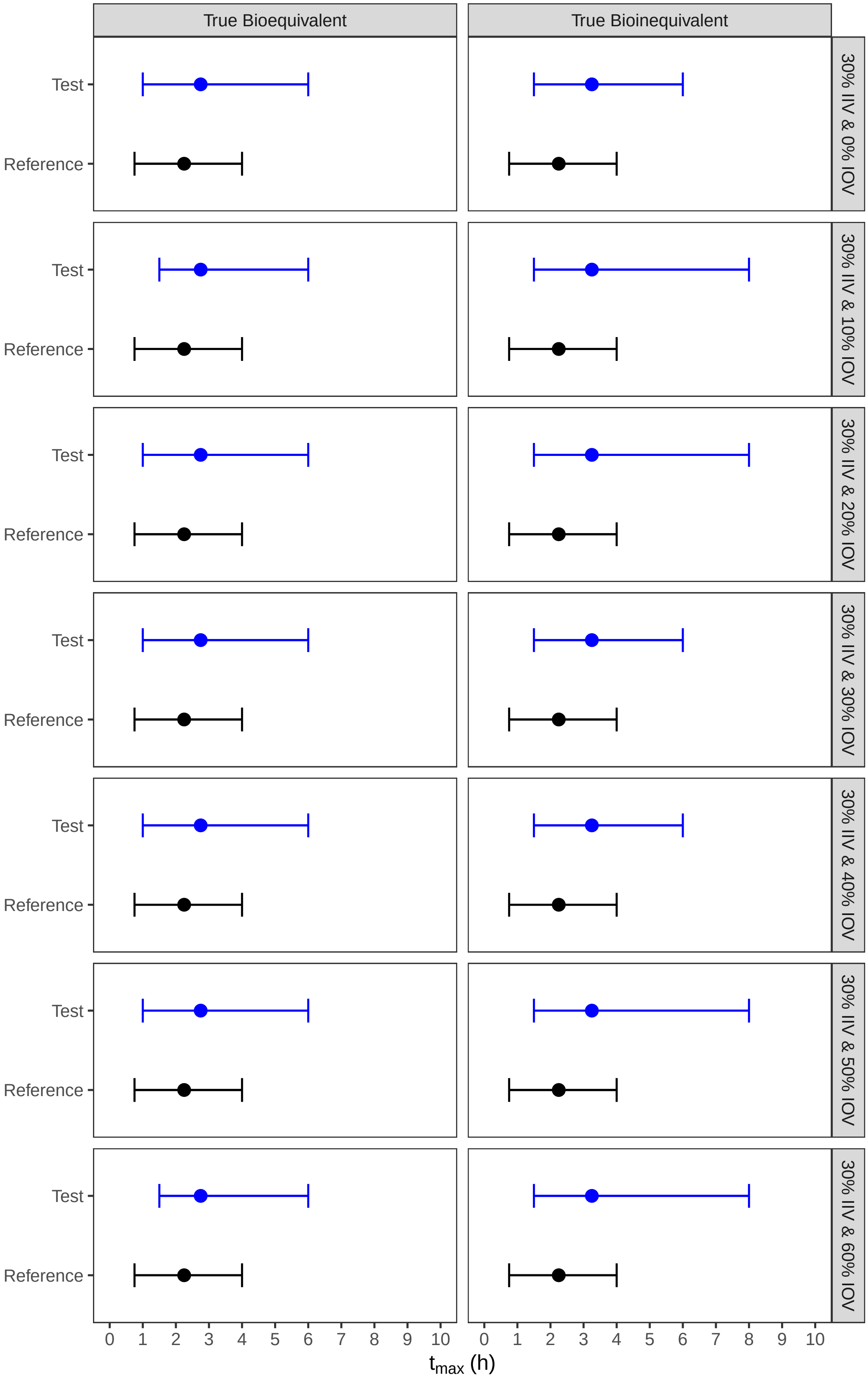

Figure S9: Distribution of Observed Tmax, for Simulations where Test Product Presents a Lower Bioavail-ability than the Reference Product

S.1.3.3. Area Under the Concentration-Time Curve

Table S4: Summary Statistics of Simulated AUC0-t, for Simulations where Test Product Presents a Lower Bioavailability than the Reference Product

	True Bioequivalent		True Bioinequivalent	
	Reference	Test	Reference	Test
30% IIV & 0% IOV	4951.25 (30.9%) [4944.86–4957.64]	4930.23 (30.9%) [4923.87–4936.6]	4961.11 (30.7%) [4954.75–4967.47]	4900.51 (30.7%) [4894.21–4906.81]
30% IIV & 10% IOV	4938.51 (32.3%) [4931.85–4945.18]	4917.09 (32.4%) [4910.46–4923.74]	4938.43 (32.6%) [4931.73–4945.14]	4877.68 (32.6%) [4871.06–4884.3]
30% IIV & 20% IOV	4961.11 (37.3%) [4953.46–4968.78]	4941.01 (37.4%) [4933.38–4948.66]	4965.32 (37.8%) [4957.56–4973.08]	4903.31 (37.8%) [4895.66–4910.98]
30% IIV & 30% IOV	4958.66 (44.5%) [4949.65–4967.69]	4932.36 (44.5%) [4923.41–4941.33]	4947.19 (44.3%) [4938.25–4956.15]	4883.78 (44.2%) [4874.96–4892.61]
30% IIV & 40% IOV	4947.1 (53.5%) [4936.5–4957.73]	4927.58 (53.3%) [4917.05–4938.13]	4946.54 (53.3%) [4935.98–4957.13]	4892.73 (53.1%) [4882.32–4903.17]
30% IIV & 50% IOV	4939.95 (63.2%) [4927.72–4952.21]	4903.76 (63.5%) [4891.57–4915.98]	4951.22 (63.6%) [4938.88–4963.58]	4893.86 (63.6%) [4881.67–4906.08]
30% IIV & 60% IOV	5001.7 (75.4%) [4987.36–5016.08]	4954.46 (75.9%) [4940.19–4968.78]	4963.79 (75.2%) [4949.6–4978.02]	4895.88 (74.9%) [4881.92–4909.87]

Values for Area Under the Concentration-Time Curve ($AUC_{0\text{-}t}$) are in μg·h/L, and are presented as G_{mean}, with GCV% within parenthesis, and 95% CI of the G_{mean} within square brackets.

$N = 210000$ for each group.

IIV – Inter-individual variability; IOV – Inter-occasion variability.

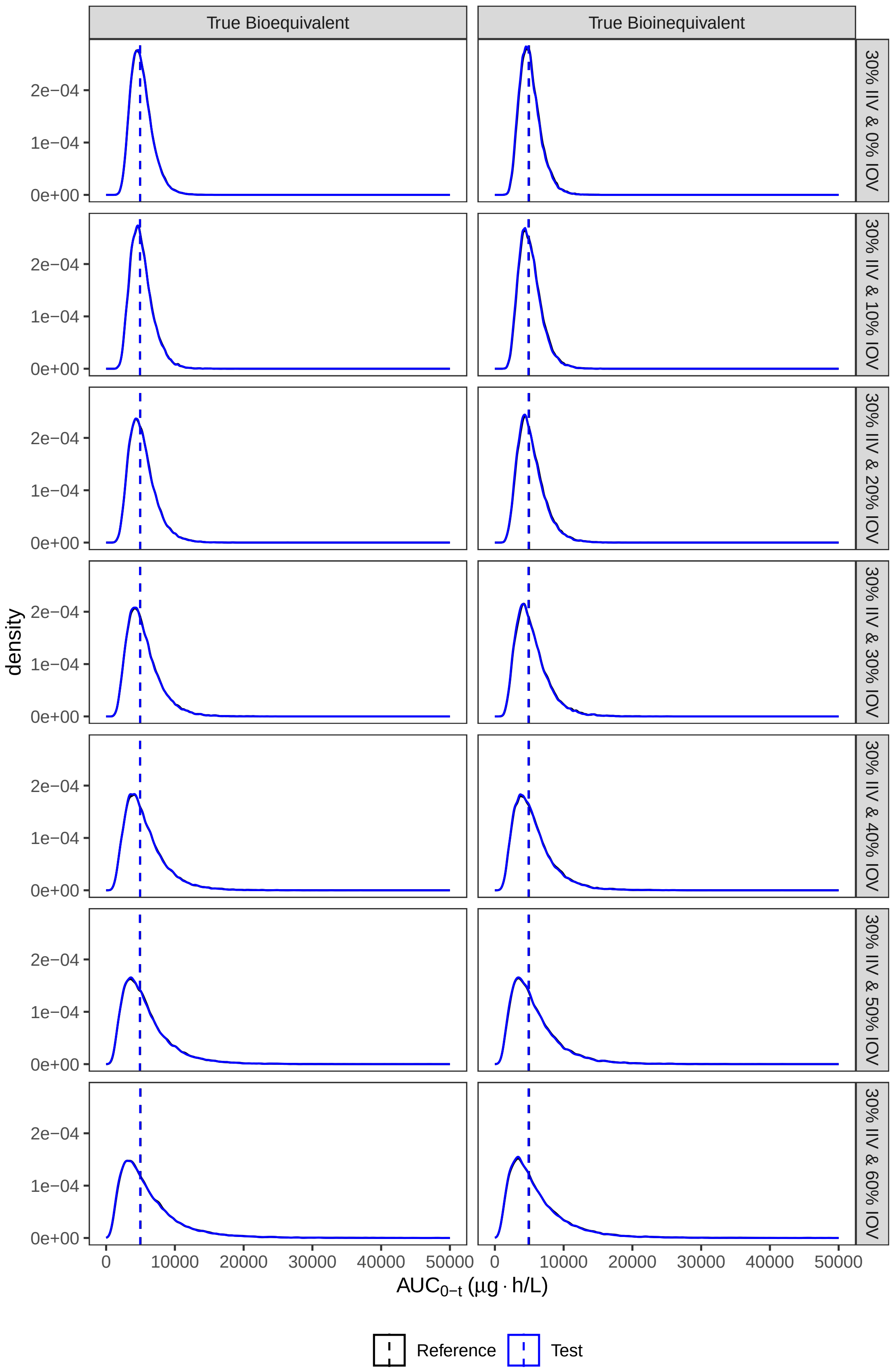

Figure S10: Distribution of Observed AUC0-t, for Simulations where Test Product Presents a Lower Bioavail-ability than the Reference Product

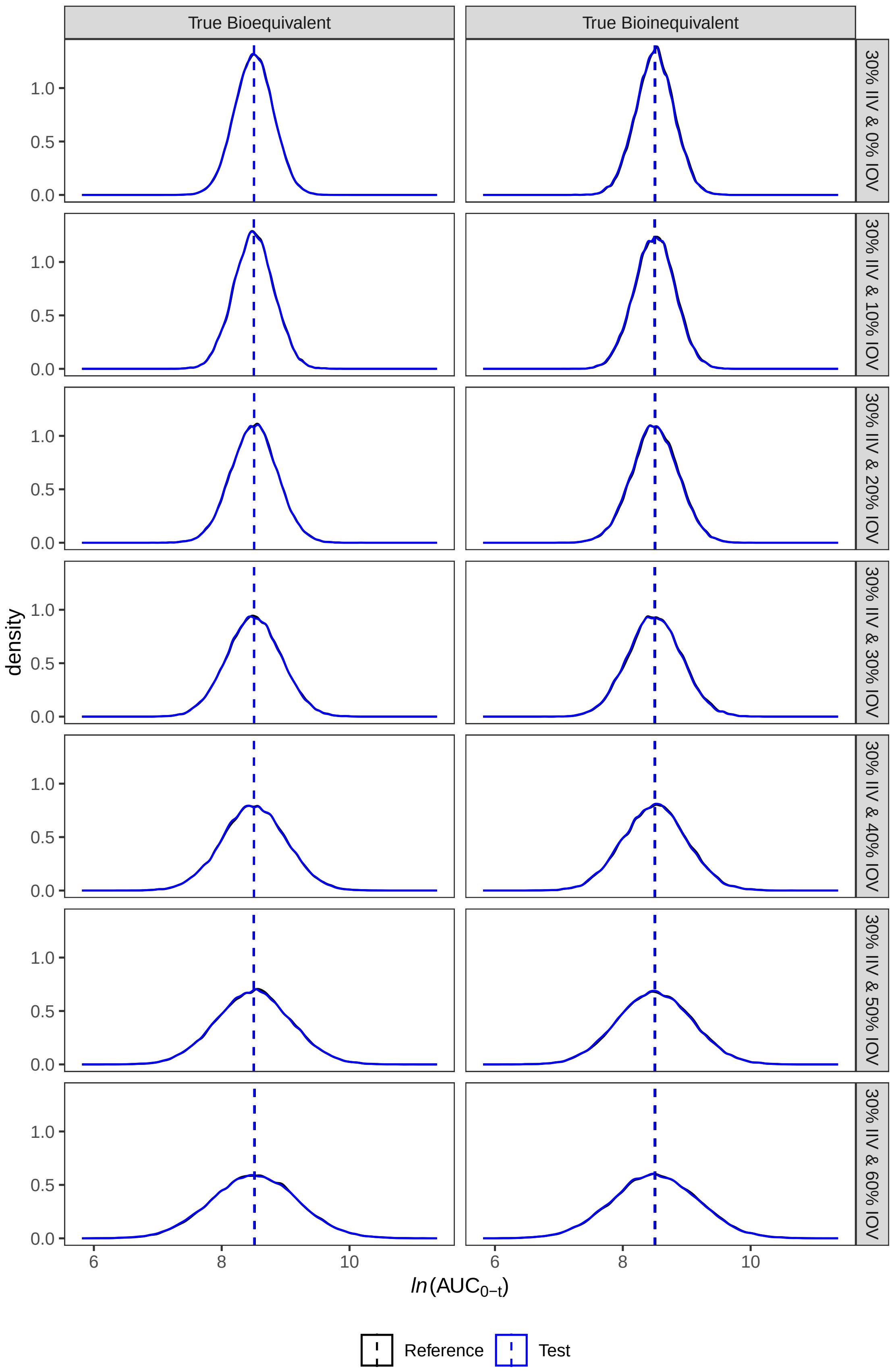

Figure S11: Distribution of *Ln*-Transformed Observed AUC0-t, for Simulations where Test Product Presents a Lower Bioavailability than the Reference Product

S.2. Simulations Where Test Product Presents a Higher Bioavailability than the Reference Product

S.2.1. Simulated Volume of Distribution

Table S5: Summary Statistics of Simulated Volume of Distribution, for Simulations where Test Product Presents a Higher Bioavailability than the Reference Product

	True Bioequivalent		True Bioinequivalent	
	Reference	Test	Reference	Test
30% IIV & 0% IOV	58.85 (30.6%) [58.77–58.92]	58.85 (30.6%) [58.77–58.92]	58.78 (30.9%) [58.7–58.86]	58.78 (30.9%) [58.7–58.86]
30% IIV & 10% IOV	58.96 (32.2%) [58.88–59.04]	58.95 (32.2%) [58.87–59.03]	58.73 (32.6%) [58.65–58.81]	58.74 (32.5%) [58.66–58.82]
30% IIV & 20% IOV	58.71 (37.1%) [58.62–58.8]	58.66 (37.1%) [58.57–58.75]	59.06 (37.4%) [58.97–59.15]	59.08 (37.4%) [58.99–59.17]
30% IIV & 30% IOV	58.9 (45%) [58.79–59.01]	58.89 (44.8%) [58.78–58.99]	58.73 (44.5%) [58.62–58.84]	58.77 (44.5%) [58.66–58.88]
30% IIV & 40% IOV	58.56 (53.1%) [58.44–58.69]	58.54 (53.2%) [58.42–58.67]	58.62 (53.1%) [58.5–58.75]	58.68 (53%) [58.56–58.81]
30% IIV & 50% IOV	58.32 (63.4%) [58.17–58.46]	58.42 (63.5%) [58.27–58.56]	58.81 (63.5%) [58.66–58.96]	58.91 (63.5%) [58.77–59.06]
30% IIV & 60% IOV	59.13 (75.1%) [58.96–59.3]	59.05 (75.1%) [58.88–59.22]	58.65 (75.8%) [58.48–58.82]	58.44 (75.9%) [58.27–58.61]

Values for V are in L, and are presented as G_{mean}, with GCV% within parenthesis, and 95% CI of the G_{mean} within square brackets.

$N = 210000$ for each group.

IIV – Inter-individual variability; IOV – Inter-occasion variability.

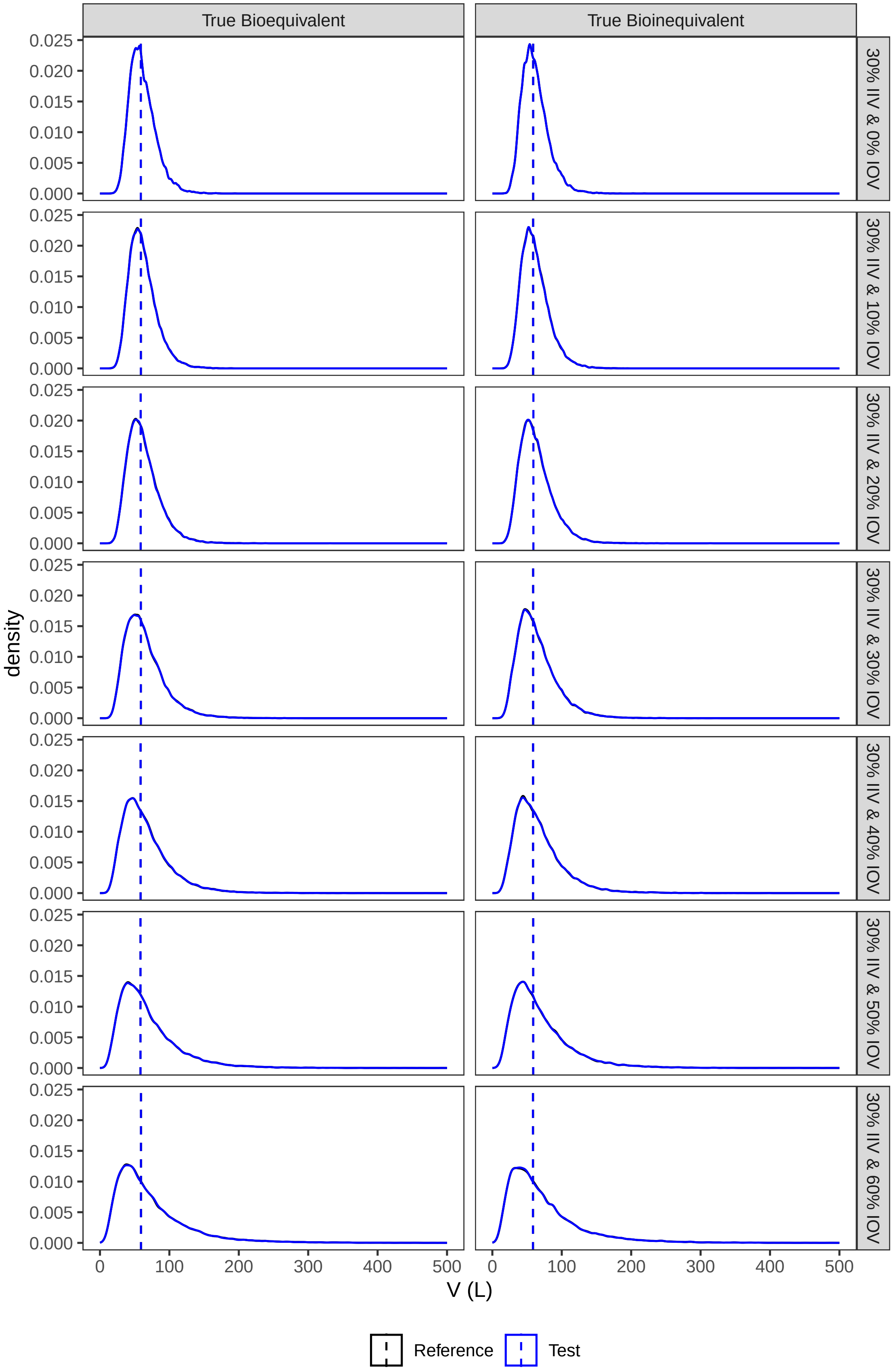

Figure S12: Distribution of Simulated Volume of Distribution, for Simulations where Test Product Presents a Higher Bioavailability than the Reference Product

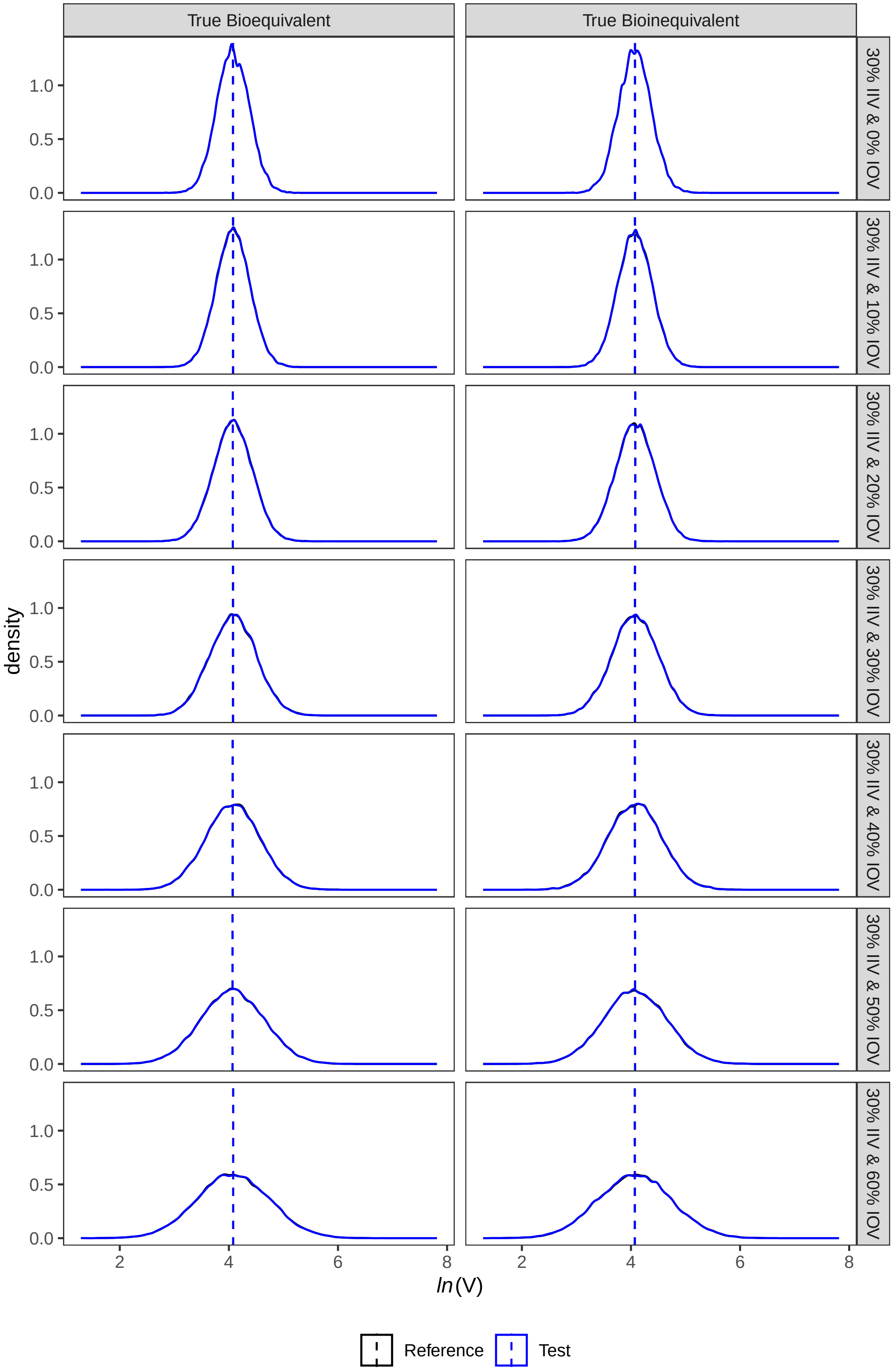

Figure S13: Distribution of *Ln*-Transformed Simulated Volume of Distribution, for Simulations where Test Product Presents a Higher Bioavailability than the Reference Product

S.2.2. Simulated Plasma Concentrations

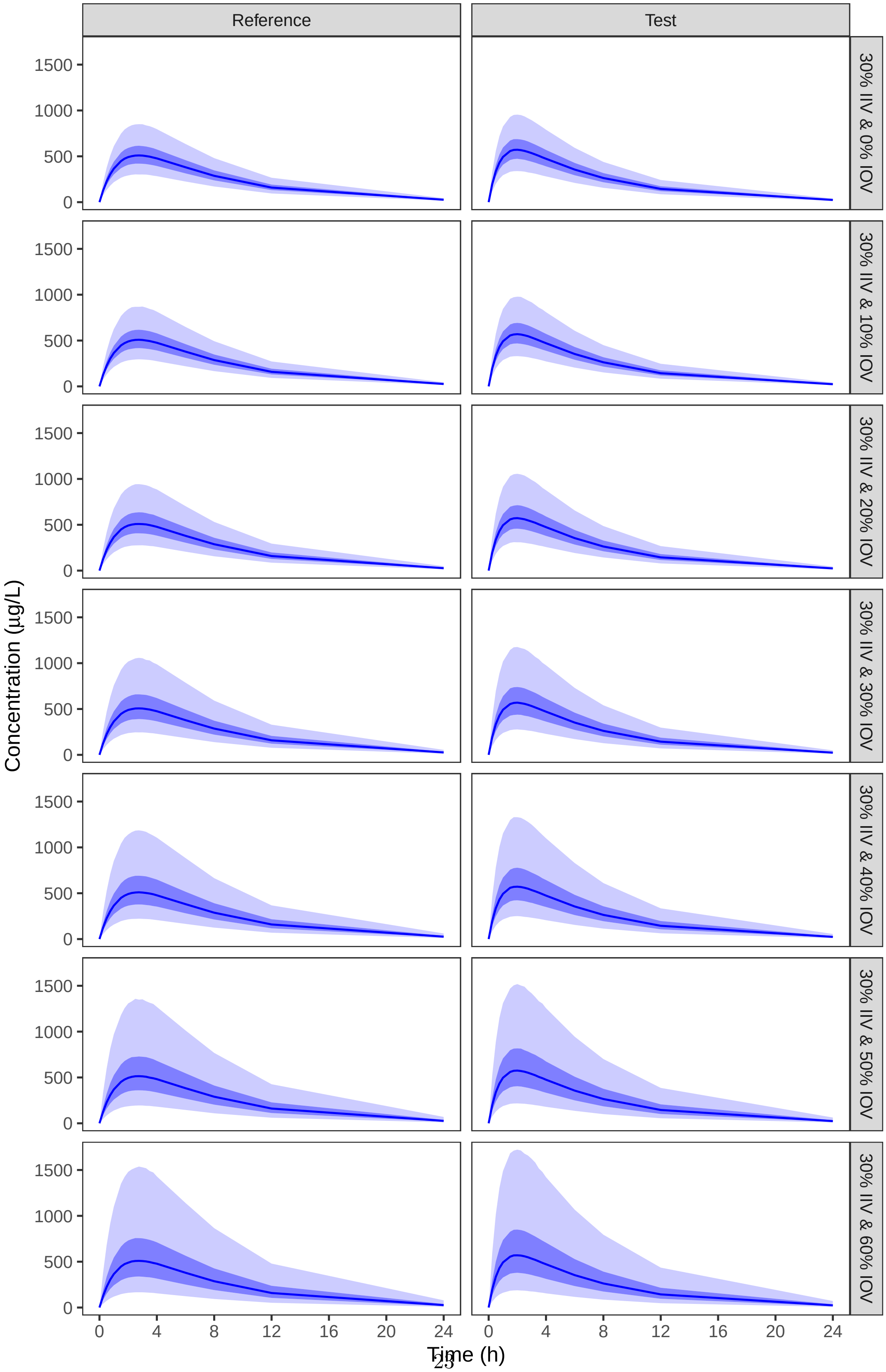

Figure S14: 90% Confidence Interval of Simulated Concentration-Time Data, for True Bioequivalent Simula-tions where Test Product Presents a Higher Bioavailability than the Reference Product – Linear Scale

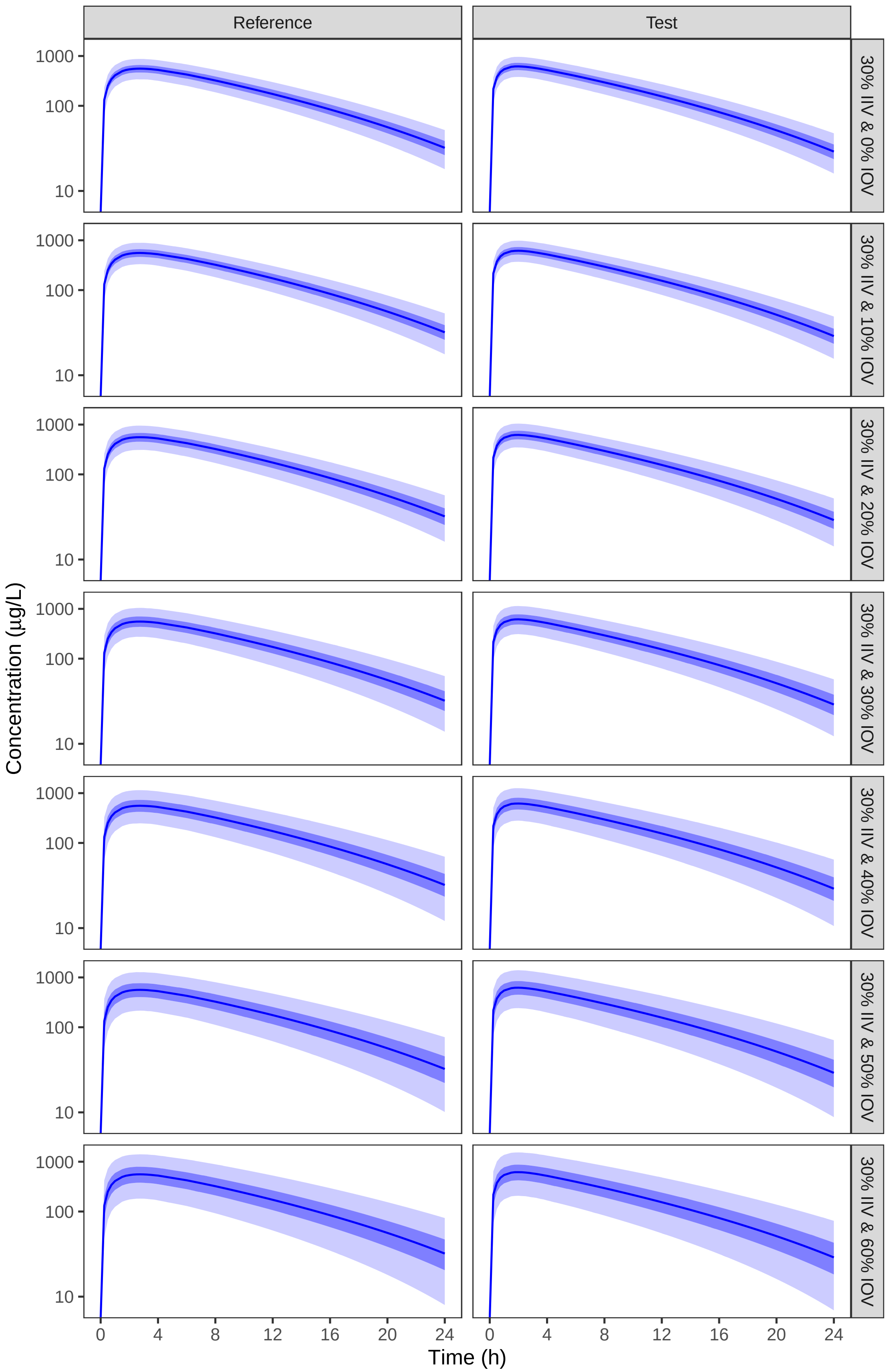

Figure S15: 90% Confidence Interval of Simulated Concentration-Time Data, for True Bioequivalent Simula-tions where Test Product Presents a Higher Bioavailability than the Reference Product – Semi-Logarithmic Scale

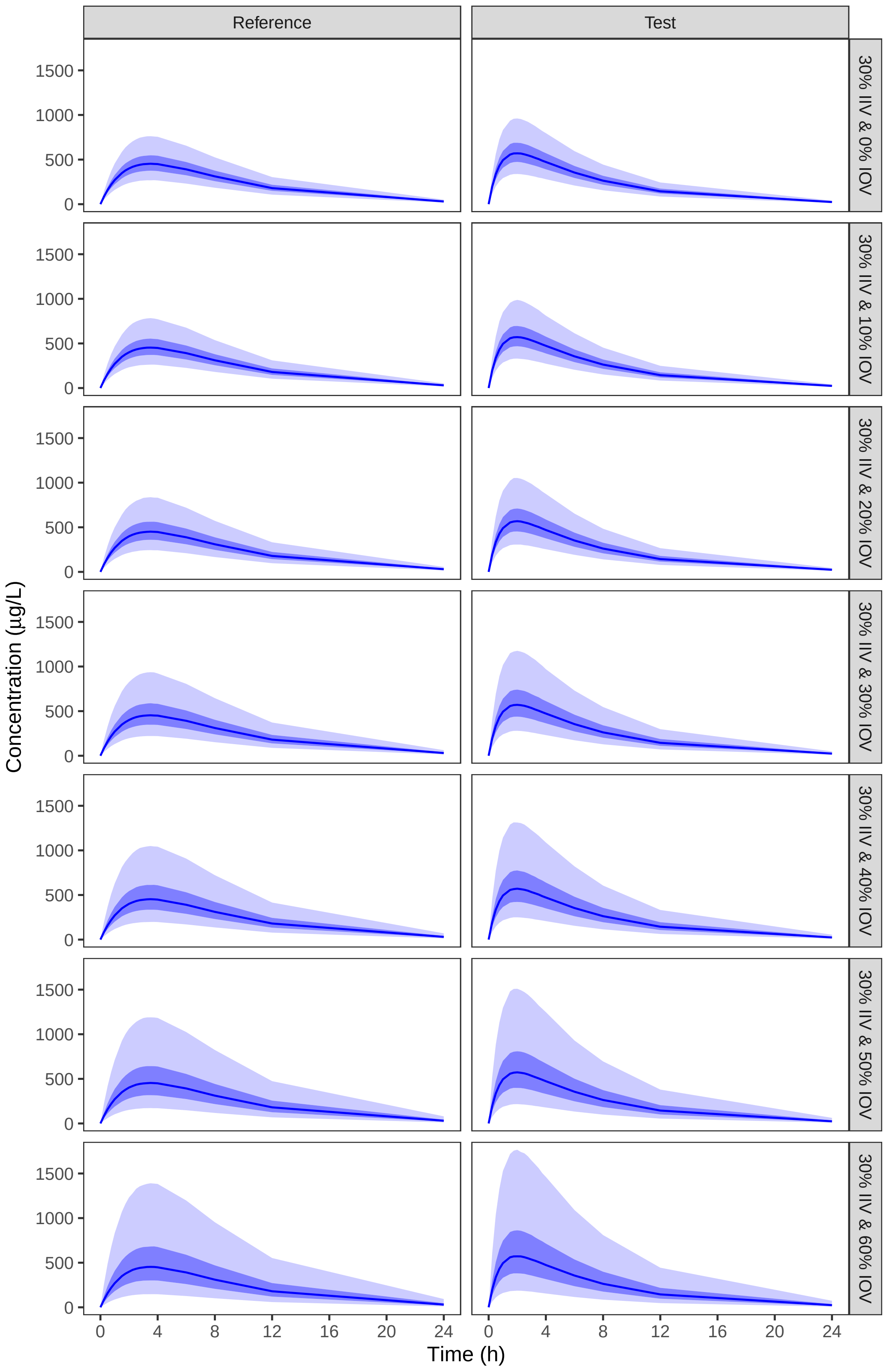

Figure S16: 90% Confidence Interval of Simulated Concentration-Time Data, for True Bioinequivalent Simulations where Test Product Presents a Higher Bioavailability than the Reference Product – Linear Scale

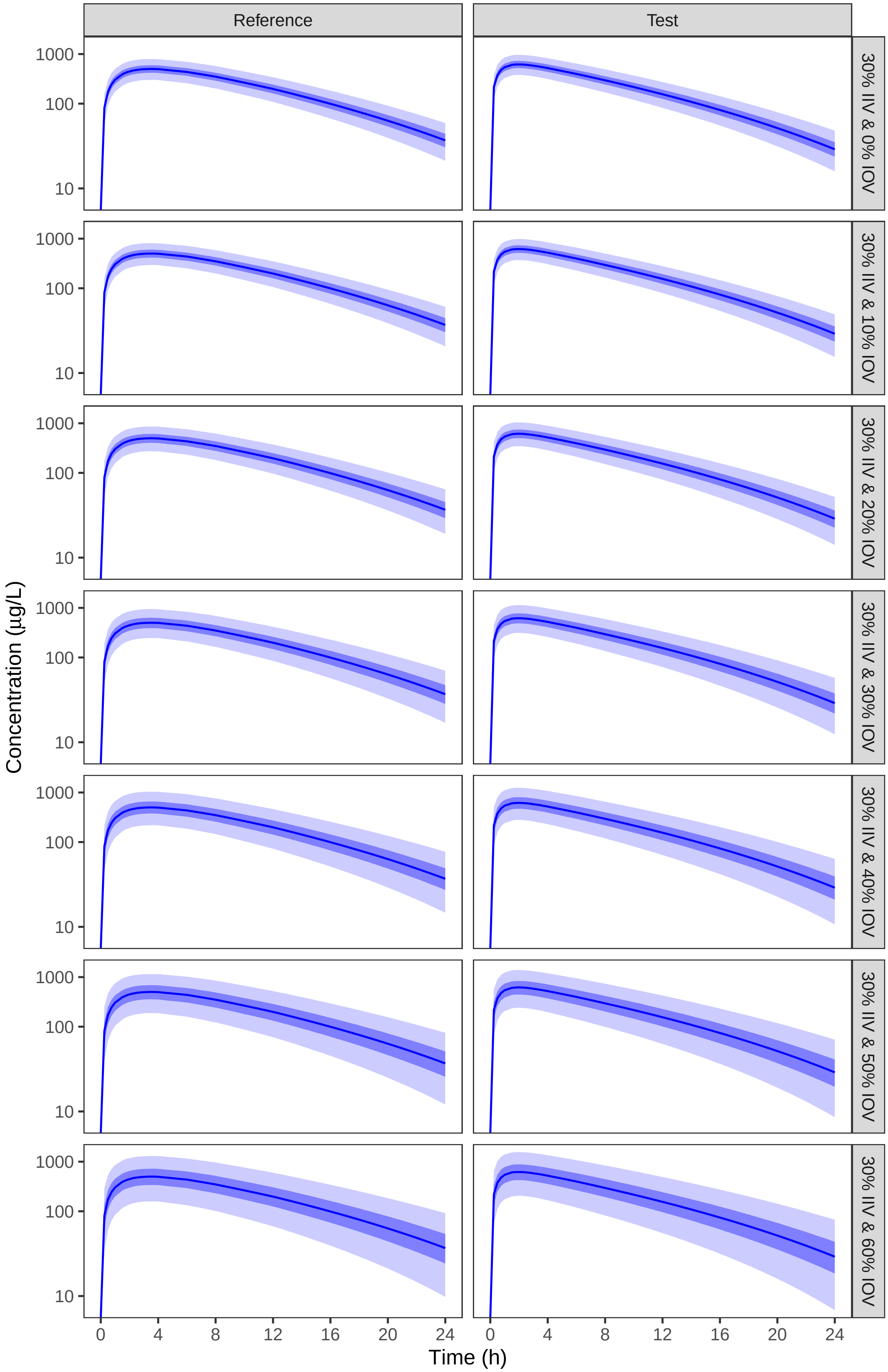

Figure S17: 90% Confidence Interval of Simulated Concentration-Time Data, for True Bioinequivalent Simu-lations where Test Product Presents a Higher Bioavailability than the Reference Product – Semi-Logarithmic Scale

S.2.3. Simulated Non-Compartmental Analysis Parameters

S.2.3.1. Maximum Observed Concentration

Table S6: Summary Statistics of Simulated Cmax, for Simulations where Test Product Presents a Higher Bioavailability than the Reference Product

	True Bioequivalent		True Bioinequivalent	
	Reference	Test	Reference	Test
30% IIV & 0% IOV	579.57 (31.2%) [578.82–580.33]	641.66 (31.3%) [640.82–642.5]	513.72 (31.6%) [513.04–514.4]	642.8 (31.6%) [641.95–643.64]
30% IIV & 10% IOV	578.73 (32.8%) [577.94–579.53]	641.22 (32.9%) [640.34–642.1]	514.2 (33.2%) [513.49–514.91]	642.82 (33.1%) [641.93–643.71]
30% IIV & 20% IOV	580.87 (37.7%) [579.97–581.78]	644.03 (37.7%) [643.03–645.04]	511.08 (37.9%) [510.28–511.88]	639.45 (37.9%) [638.44–640.45]
30% IIV & 30% IOV	578.83 (45.5%) [577.76–579.91]	641.05 (45.3%) [639.86–642.23]	513.89 (45%) [512.95–514.84]	642.57 (45%) [641.39–643.76]
30% IIV & 40% IOV	582.6 (53.5%) [581.35–583.85]	645.29 (53.6%) [643.9–646.67]	514.9 (53.6%) [513.79–516.01]	643.21 (53.4%) [641.83–644.59]
30% IIV & 50% IOV	584.74 (63.8%) [583.28–586.2]	646.39 (63.9%) [644.78–648.01]	513.59 (63.9%) [512.3–514.87]	641.53 (63.9%) [639.92–643.14]
30% IIV & 60% IOV	577.22 (75.5%) [575.56–578.88]	639.92 (75.5%) [638.08–641.76]	514.87 (76.1%) [513.39–516.36]	646.34 (76.3%) [644.47–648.22]

Values for C_{max} are in μg/L, and are presented as G_{mean}, with GCV% within parenthesis, and 95% CI of the G_{mean} within square brackets.

$N = 210000$ for each group.

IIV – Inter-individual variability; IOV – Inter-occasion variability.

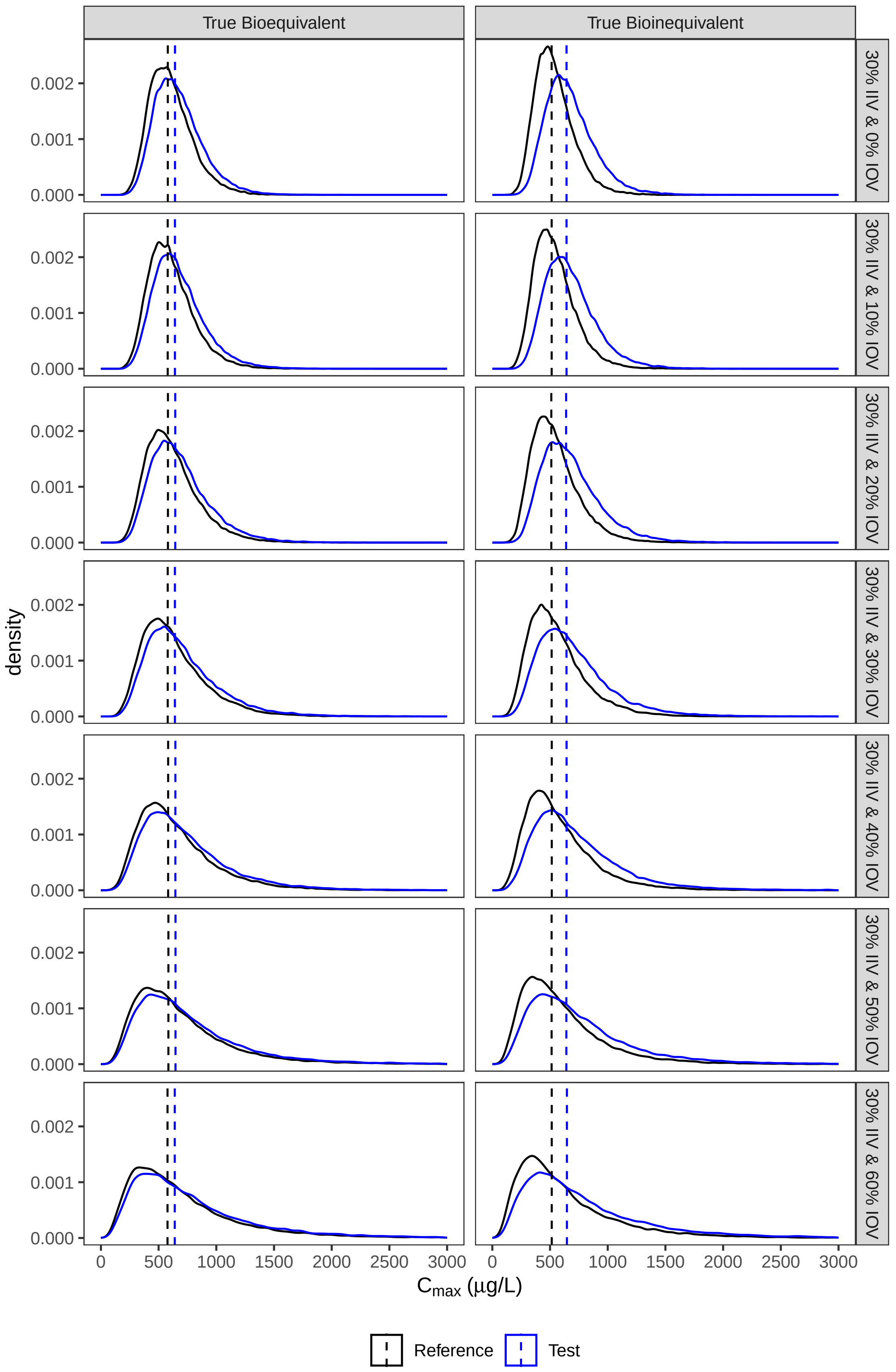

Figure S18: Distribution of Observed Cmax, for Simulations where Test Product Presents a Higher Bioavail-ability than the Reference Product

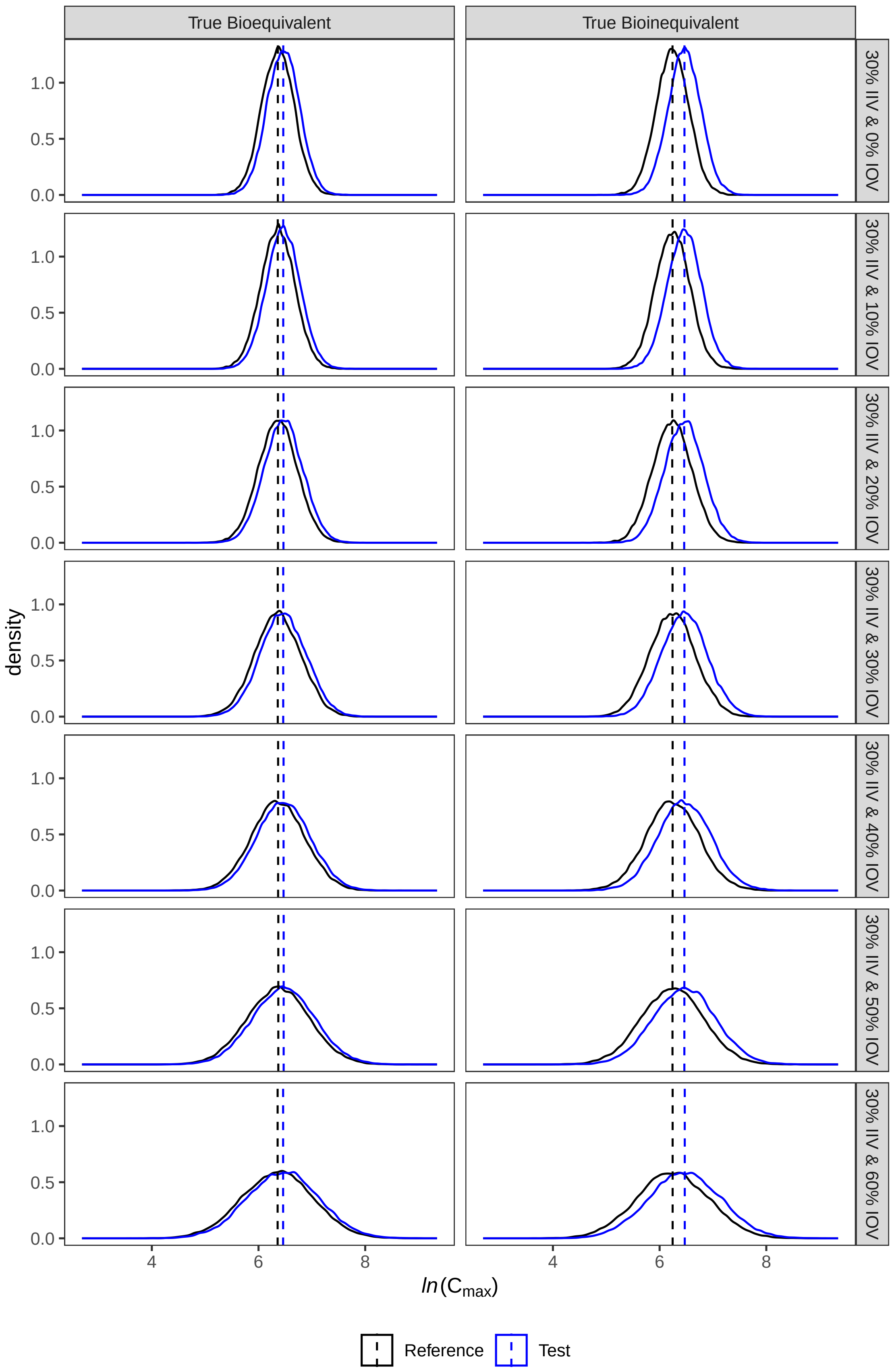

Figure S19: Distribution of *Ln*-Transformed Observed Cmax, for Simulations where Test Product Presents a Higher Bioavailability than the Reference Product

S.2.3.2. Time of Maximum Observed Concentration

Table S7: Summary Statistics of Simulated Cmax, for Simulations where Test Product Presents a Higher Bioavailability than the Reference Product

	True Bioequivalent		True Bioinequivalent	
	Reference	Test	Reference	Test
30% IIV & 0% IOV	2.75 (1–6)	2.25 (0.75–4)	3.25 (1.5–6)	2.25 (0.75–4)
30% IIV & 10% IOV	2.75 (1.5–6)	2.25 (0.75–4)	3.25 (1.5–6)	2.25 (0.75–4)
30% IIV & 20% IOV	2.75 (1–6)	2.25 (0.75–4)	3.25 (1.5–6)	2.25 (0.75–4)
30% IIV & 30% IOV	2.75 (1–4)	2.25 (0.75–4)	3.25 (1.5–6)	2.25 (0.75–4)
30% IIV & 40% IOV	2.75 (1–6)	2.25 (0.75–4)	3.25 (1.5–8)	2.25 (1–4)
30% IIV & 50% IOV	2.75 (1.5–6)	2.25 (0.75–4)	3.25 (1.5–6)	2.25 (0.75–4)
30% IIV & 60% IOV	2.75 (1–6)	2.25 (0.75–4)	3.25 (1.5–6)	2.25 (0.75–4)

Values for t_{max} are in hours (h), and are presented as median with range within parenthesis.

$N = 210000$ for each group.

IIV – Inter-individual variability; IOV – Inter-occasion variability.

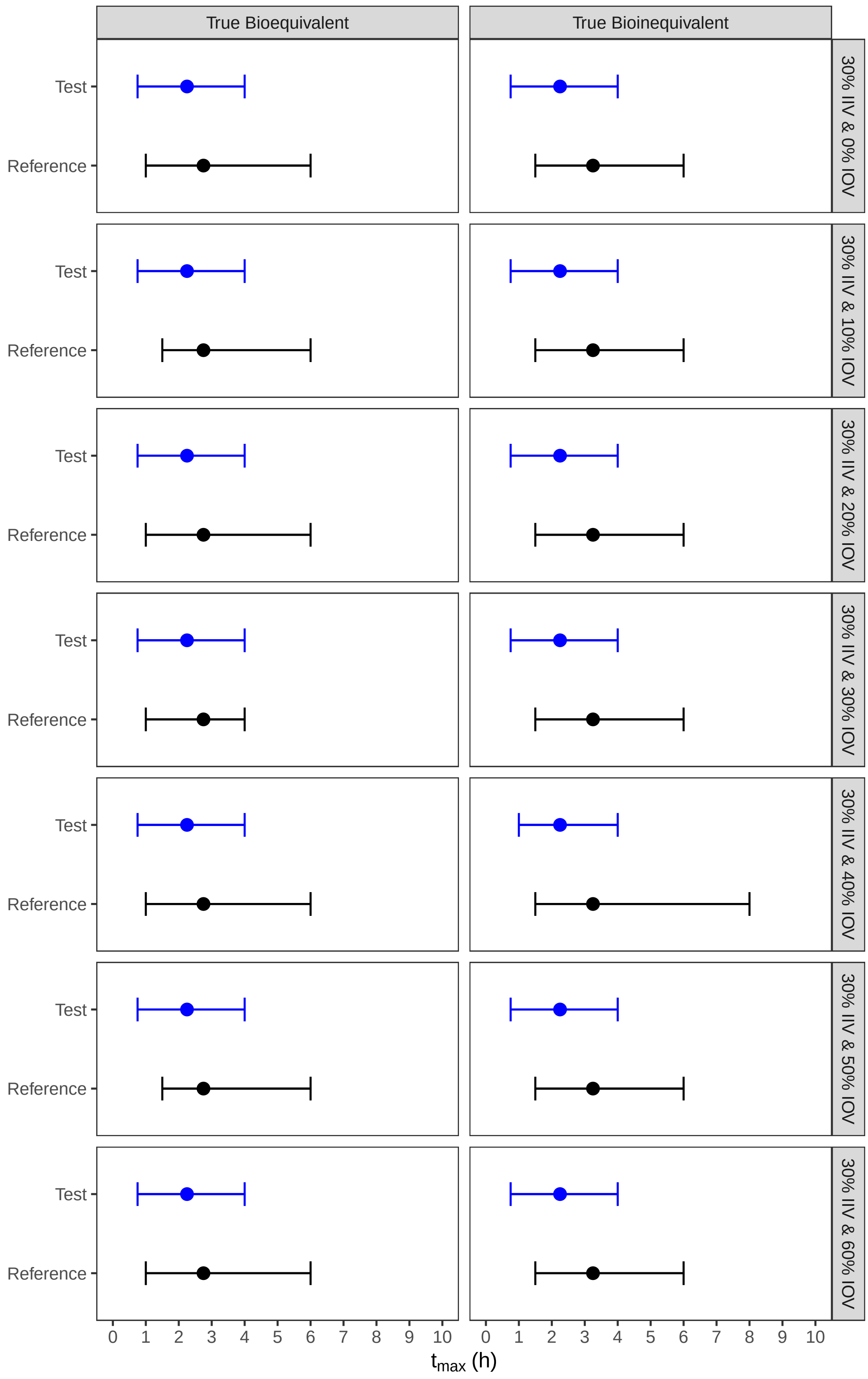

Figure S20: Distribution of Observed Tmax, for Simulations where Test Product Presents a Higher Bioavail-ability than the Reference Product

S.2.3.3. Area Under the Concentration-Time Curve

Table S8: Summary Statistics of Simulated AUC0-t, for Simulations where Test Product Presents a Higher Bioavailability than the Reference Product

	True Bioequivalent		True Bioinequivalent	
	Reference	Test	Reference	Test
30% IIV & 0% IOV	4924.2 (30.8%) [4917.87–4930.54]	4946.53 (30.8%) [4940.17–4952.89]	4891.58 (31.1%) [4885.22–4897.95]	4952.6 (31.1%) [4946.17–4959.03]
30% IIV & 10% IOV	4915.98 (32.4%) [4909.34–4922.62]	4938.35 (32.4%) [4931.69–4945.03]	4895.03 (32.8%) [4888.35–4901.73]	4953.92 (32.7%) [4947.16–4960.68]
30% IIV & 20% IOV	4935.43 (37.3%) [4927.82–4943.05]	4961.53 (37.3%) [4953.88–4969.19]	4867.23 (37.6%) [4859.67–4874.81]	4926.92 (37.6%) [4919.27–4934.58]
30% IIV & 30% IOV	4921.22 (45.2%) [4912.16–4930.3]	4943.32 (44.9%) [4934.26–4952.4]	4895.59 (44.7%) [4886.66–4904.53]	4953.01 (44.7%) [4943.98–4962.06]
30% IIV & 40% IOV	4949.14 (53.2%) [4938.58–4959.72]	4972.44 (53.3%) [4961.82–4983.09]	4904.24 (53.3%) [4893.76–4914.73]	4959.13 (53.1%) [4948.58–4969.71]
30% IIV & 50% IOV	4969.5 (63.6%) [4957.13–4981.9]	4982.45 (63.6%) [4970.04–4994.89]	4888.86 (63.6%) [4876.68–4901.07]	4941.64 (63.6%) [4929.33–4953.97]
30% IIV & 60% IOV	4900.56 (75.2%) [4886.54–4914.62]	4927.91 (75.3%) [4913.82–4942.05]	4902.43 (75.8%) [4888.31–4916.58]	4981.22 (75.9%) [4966.86–4995.61]

Values for $AUC_{0\text{-}t}$ are in μg·h/L, and are presented as G_{mean}, with GCV% within parenthesis, and 95% CI of the G_{mean} within square brackets.

$N = 210000$ for each group.

IIV – Inter-individual variability; IOV – Inter-occasion variability.

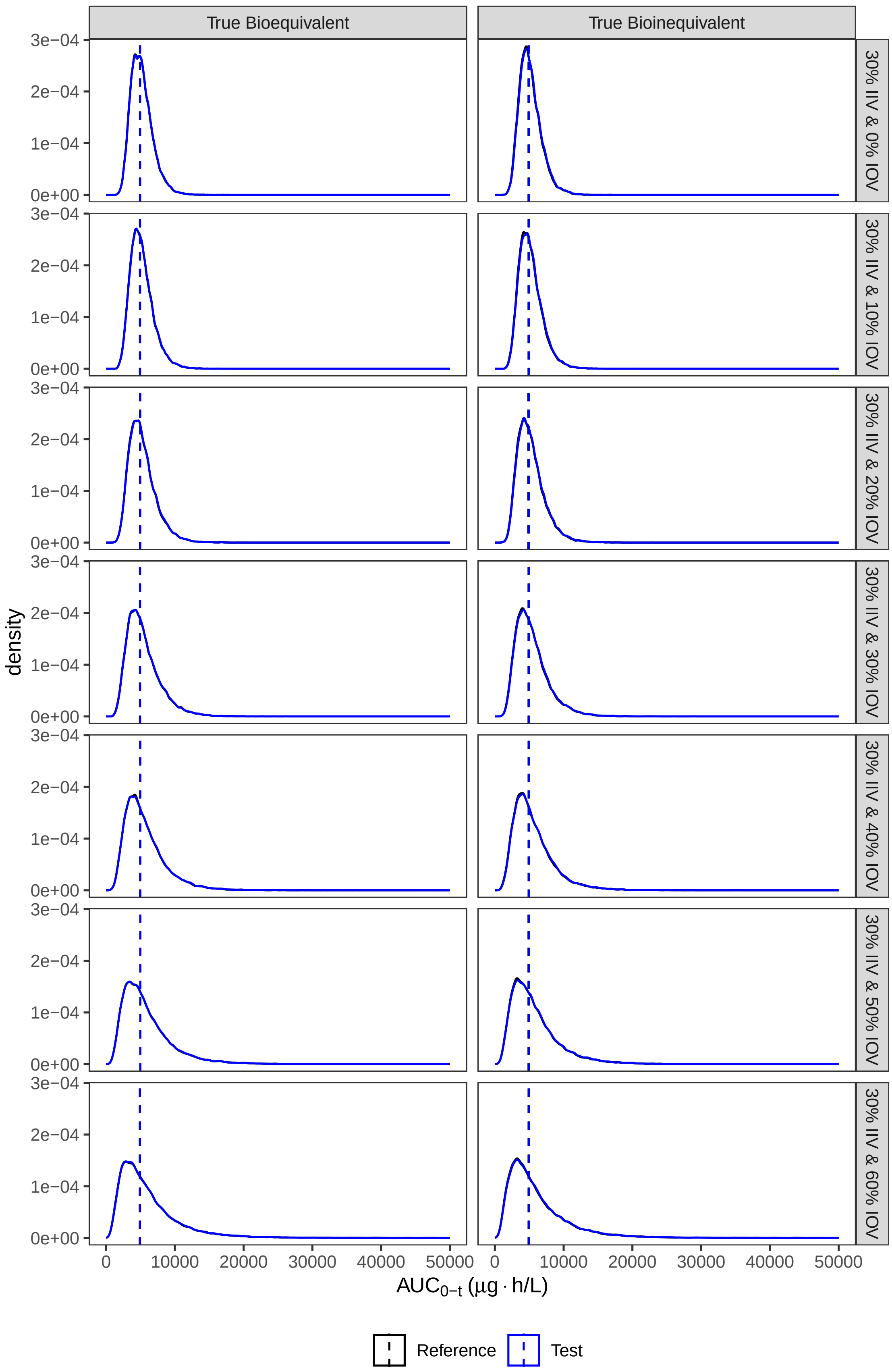

Figure S21: Distribution of Observed AUC0-t, for Simulations where Test Product Presents a Higher Bioavail-ability than the Reference Product

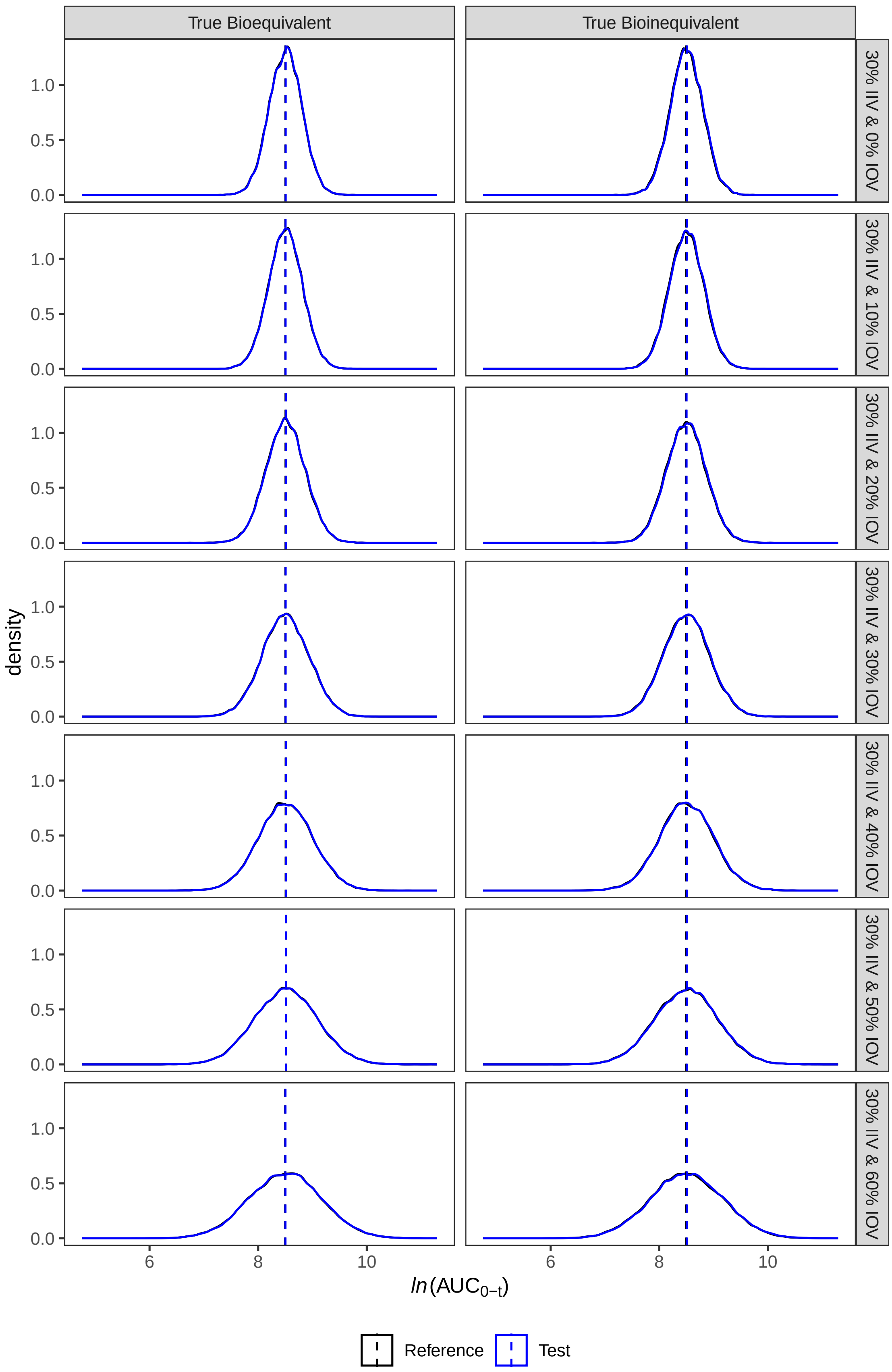

Figure S22: Distribution of *Ln*-Transformed Observed AUC0-t, for Simulations where Test Product Presents a Higher Bioavailability than the Reference Product
